# Microbial Fuel Cell stack performance enhancement through carbon veil anode modification with activated carbon powder

**DOI:** 10.1016/j.apenergy.2019.114475

**Published:** 2020-03-15

**Authors:** Iwona Gajda, John Greenman, Ioannis Ieropoulos

**Affiliations:** a Bristol BioEnergy Centre, Bristol Robotics Laboratory, University of the West of England, Bristol BS16 1QY, UK; b Department of Applied Sciences, University of the West of England, Bristol BS16 1QY, UK

**Keywords:** Activated carbon, Ceramic, Long-term operation, Microbial Fuel Cell, Urine, Stacking

## Abstract

The chemical energy contained in urine can be efficiently extracted into direct electricity by Microbial Fuel Cell stacks to reach usable power levels for practical implementation and a decentralised power source in remote locations. Herein, a novel type of the anode electrode was developed using powdered activated carbon (PAC) applied onto the carbon fibre scaffold in the ceramic MFC stack to achieve superior electrochemical performance during 500 days of operation. The stack equipped with modified anodes (MF-CV) produced up to 37.9 mW (21.1 W m^−3^) in comparison to the control (CV) that reached 21.4 mW (11.9 W m^−3^) showing 77% increase in power production. The novel combination of highly porous activated carbon particles applied onto the conductive network of carbon fibres promoted simultaneously electrocatalytic activity and increased surface area, resulting in excellent power output from the MFC stack as well as higher treatment rate. Considering the low cost and simplicity of the material preparation, as well as the outstanding electrochemical activity during long term operation, the resulting modification provides a promising anode electrocatalyst for high-performance MFC stacks to enhance urine and waste treatment for the purpose of future scale-up and technology implementation as an applied off-grid energy source.

## Highlights

• Novel modification of carbon veil anode with activated carbon in urine fed MFC stack.
• Modified microstructure enhanced surface area and power performance up to 21.1 W m^−3^.
• The modification exhibited 77% increase in power output of the MFC stack.
• It improved COD reduction by 13.5% in comparison to control stack.
• It exhibited a stable performance after 500 days and resilience to prolonged starvation.

## Graphical abstract

**Figure:**
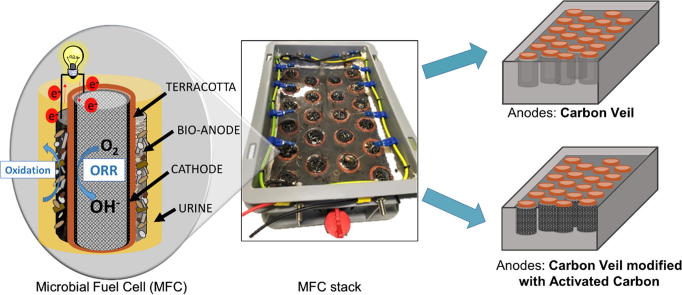

## 1 Introduction

Microbial Fuel Cell is a type of bioelectrochemical device that employs electroactive microorganisms to transform chemical energy into direct electricity by means of a combination of microbial metabolic and electrochemical reactions from a variety of substrates [1] including wastewater [2] and urine [3]. In the anode of the MFCs, microbial consortia break down organic “fuel” into electrons and protons. Electrons derived from the anaerobic oxidation by the microbial consortia are transported to the anodic electrode and travel through the external circuit to the cathode electrode, while protons and other cations pass through a membrane, which separates both chambers. Urine is a valuable “waste” that could potentially be used to off-set the need to mine and produce synthetic fertilisers [4]. It is also an energy-rich substrate possessing good electrical conductivity due to high ionic content, which makes it an ideal substrate for the production of direct electric current as well as nutrient recovery [5] both in MFCs [6] and Microbial Electrolysis Cells (MECs) [7]. Urine operated MFCs have the ability to generate electricity in remote locations as the off- grid energy source and can thus be used in areas of poor energy infrastructure. There are however major limiting factors that hinder the practical implementation of MFCs at larger scale, which are low power output, material cost and difficulties in the scale-up process as well as system longevity. Multiple findings suggest that the viability of the urine-operated systems is directly correlated with its use [8], [9] and its suitability for generating enough power to directly light an LED [10] or charge a mobile phone [11]. Technology scale-up is of enormous importance as it brings the bioelectrochemical technology innovations into real world environments, where it is tested in practice for electricity production [8], [9], treatment [12] and hydrogen generation [13]. One approach into scale-up is through stacking and multiplication of MFC units [14] where the miniaturisation of stacked [15] reactors achieves higher energy efficiency through lower ohmic losses [14]. To improve the MFC output, efforts have been made into the reactor design focusing on a membrane-based [15] or membrane-less configuration [9], [16], anodic [17] and cathodic modifications [18], [19] as well as achieving low-cost and low-tech production procedures for materials including ceramic [20] and cardboard [21]. Numerous anode modifications have been pursued over the past decades looking into the metallic [22], carbon [23], composite [24] and chemical modifications [25] that aim to increase its specific surface area as well as to increase electron transfer rates, however in light of the practical application of the technology, selected materials and methodology of their synthesis/preparation should take cost and ease of assembly into account [26] in order to allow for the electrode materials to be mass produced in a cost-effective way. However, the vast majority of electrode modification strategies are unsuitable for practical applications due to complex manufacturing processes and high costs, and their long-term stability is rarely tested [27]. One of the most promising, readily available and affordable materials is activated carbon [28] typically used in granular (GAC) form in the anodic half-cell [29] and powdered form (PAC) for the fabrication of the cathode when applied onto stainless steel mesh [9], [30] or carbon matrix [10], [31]. PAC-based cathodes are widely applied in MFC studies including field trials and prototypes of the membrane-based [8] and membrane-less systems [9] and its properties suggest it would be an excellent candidate to adopt in the biotic anode because of the micro-nanostructrure of the activated carbon powder particles. So far, anodes with nanoscale characteristics have been developed including carbon nanotubes [32], carbon black [33] and graphene [23] which promotes electricity generation in MFCs thanks to the increased surface area, improved conductivity and enhanced resultant biofilm formation. Nevertheless, the fabrication cost of these materials is relatively expensive, impeding the MFC progress into large-scale applications. Therefore, the implementation of activated carbon is an environmentally friendly and cost-effective method for the anode surface enhancement. For example, the mesoporous-microporous structure of activated carbon derived from the chestnut shell was proven suitable in the MFC anode [34] or as an addition to the constructed wetland MFC [35]. Further development of new, efficient and cost effective materials is needed for construction of sustainable bioelectrochemical technologies, which can be deployed in wastewater treatment plants and in remote locations as the decentralised power sources. This work aims to explore for the first time, simple and inexpensive modification of the anodic carbon veil scaffold with the addition of powdered activated carbon onto the fibres to produce a three-dimensional (3D) structure in order to improve the output of a MFC stack. It aims to test the MFC performance, as a result of this modification, over a long term period (500 days) under feeding and starvation regimes and discusses scale-up challenges for wider implementation. This is in order to aid the development of an affordable system that is designed for the ease of mass production of future urine-powered systems deployed as decentralised energy sources in a wide range of real world environments. In order for MFCs to be fully realised as off-the-grid power sources, research must focus on producing low cost and easy to manufacture electrode and membrane materials, which have been tested in realistic environments.

## 2 Materials and methods

### 2.1 Experimental set-up

Individual MFCs were assembled using terracotta cylinders (Jain Scientific Suppliers, India) as previously described [36]. The cylinder dimensions were 50 mm (h), 22 mm inside and 30 mm outside diameter. The cathodes were made of activated carbon (G Baldwins and Co., UK) and 20% PTFE blend applied onto the hydrophobic carbon veil as previously described [31]. The cathodes (22.5 cm^2^) were placed in the inner chamber of the cylinder, with the activated carbon layer exposed to the ceramic wall using stainless steel crocodile clips as connectors to the external circuit and data acquisition hardware. Blocks of activated carbon foam (Finest Filters, UK) were inserted into the cylinders to maintain good electrical contact between the cathode and the ceramic membrane.

For the test, two types of anode materials were prepared: carbon veil fibre (CV) as the control and modified carbon veil (MF-CV) with activated carbon powder. The preparation is described as follows:

CV- Carbon veil fibre was purchased from PRF Composites, UK with the carbon loading of 20 g/m^−2^ and cut to the dimensions of 200 × 140 mm. It was folded and wrapped around the terracotta cylinder and tightened with stainless steel wire to hold the electrode in place and to maintain an electrical connection for the external circuit.

MF-CV- identical piece of carbon veil used as the control was coated with activated carbon ink. For the preparation of the ink, food-grade powdered activated carbon originating from coconut shells, was purchased from a health store (G Baldwins and Co., UK) and had a typical surface area of 1 g between 800–1000 m^2^ and size: approx 100 µm. It was blended with 5% PTFE (60% water dispersion in H_2_O, Sigma Aldrich) and 300 mL of deionised water. The mixture was stirred for 2 min -in order to obtain a slurry and applied onto both sides of carbon veil using a paintbrush. The sheets of such prepared material were then heat treated at 250 °C for 30 min. The final loading of the activated carbon was 5 mg/cm^−2^. The modified material was folded and wrapped around the terracotta cylinder and tightened with stainless steel wire the same way as the control. Subsequently, small incisions were made towards the centre of the wrapped electrode making 4 mm gaps but preserving the continuity of the carbon fibre.

**Fig. 1:**
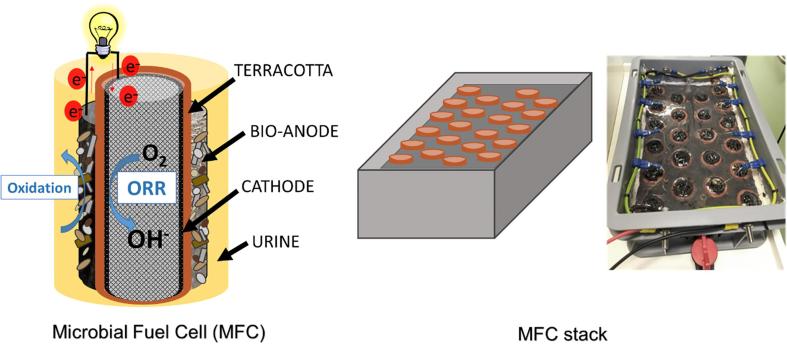
Scheme of the individual MFC and the 22 MFC units stack. Photograph of the assembled stack with parallel electrical connections.

Two MFC-stacks were assembled using Euro stacking containers (Plastor, UK) of the dimensions 300 × 200 × 118 mm and the total capacity of 5L. The container was used as the chassis with an inlet and an outlet to allow the electrolyte to flow. 22 MFC units were installed in each module (Fig. 1), equipped with modified (MF-CV) and unmodified anodes (CV) making the total volume of the anodic chamber of 1.8L. The MFCs were arranged in four rows where all the anodes and all the cathodes were electrically connected in parallel. Sealed acrylic sheet was used as a lid to fix the cylinders in position with all the anode wires connected underneath to the main anodic connection of the module. All the cathodes were connected above the lid using stainless steel wires and two cables on the sides of the module connected towards the main cathodic connection of the module (Fig. 1).

### 2.2 Operation of MFC stacks and analysis

Anaerobic activated sludge, obtained from the Wessex Water wastewater treatment plant in Saltford, UK, was mixed 1:1 with urine and used as the inoculum for both modules. Urine was collected anonymously from healthy volunteers, pooled together and stored in a 40L collection tank (pH 8.9–9.2), at room temperature and used directly as the feedstock for the MFC modules in batch mode by daily replacement.

Two different anode materials were electrochemically characterised using linear sweep voltammetry (LSV). LSVs were performed using SP-50 potentiostat (Bio-Logic), in a three-electrode configuration consisting of: Ag/AgCl 3 M (KCl) as reference electrode (inserted into the anode chamber), the tested anode as working electrode, and the MFC cathode as the counter electrode. LSVs were performed at a scan rate of 0.25 mV s^−1^. Also the cathode electrodes were studied through LSV technique. In this case, the working electrode was the MFC cathode, the counter electrode was the anode and the reference electrode was Ag/AgCl 3 M (KCl). Polarisation curves were obtained by plotting voltage against current. The power generated was obtained by multiplying voltage and current values and the power curves were plotted against current. The Chemical Oxygen Demand (COD) consumption by the modules was monitored where samples were filtered using Nylon filters, 0.45 µm pore size prior to the measurement. COD analysis was carried out using colorimetric method using the potassium dichromate (COD HR, Jenway, UK) and a MD 200 photometer (Lovibond, UK). Soluble COD removal (%) was determined as the difference between the concentration at the beginning and the end of a treatment cycle.

## 3 Results and discussion

### 3.1 Anode surface morphology

**Fig. 2:**
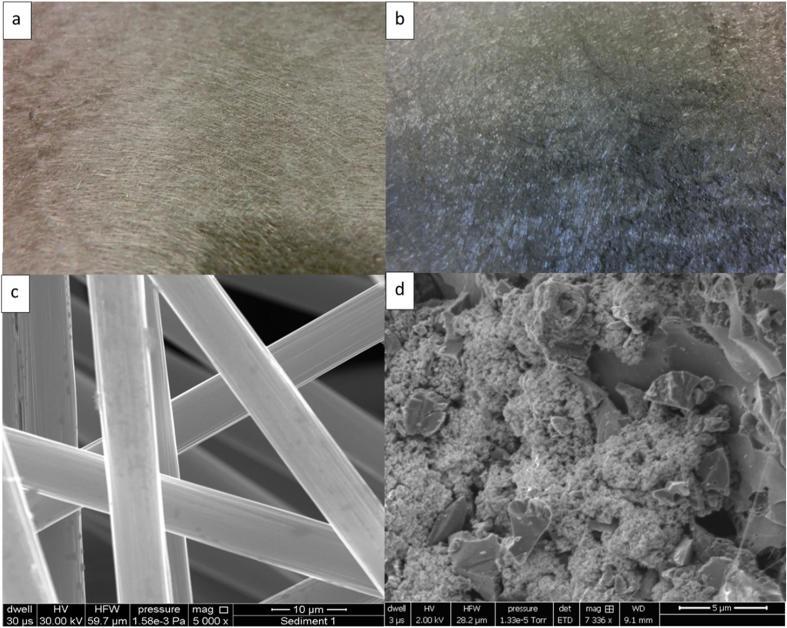
(a) Carbon veil fibre (CV), (b) modified carbon veil with activated carbon particles (MF-CV), (c) carbon veil under 5000× magnification, (d) modified carbon veil under 7336× magnification.

Surface morphology of the carbon fibre veil material assembled as the control anode electrode showed uniform network of carbon fibres that are continuous and interconnected with macropores in between the cylindrical fibres (Fig. 2a and 2c), however the surface of the individual fibres is smooth and non-porous (Fig. 2c). Anodic modification with the activated carbon powder showed additional layers of clustered particles of activated carbon attached to the surface of carbon fibres as well as their presence in between the fibres, partially filling up the gaps (Fig. 2d). The SEM image clearly indicates the microporous structure of the modification with the PTFE binder scattered in between the porous activated carbon. The carbon veil fibres (Fig. 2b and d) holding the microporous structure work as a scaffold and a current collection layer for the electrons.

Although this modification was handmade and the distribution of the particles was not uniform, the particles of activated carbon were able to adhere to the carbon veil matrix thanks to the presence of PTFE. PTFE and activated carbon mixture is typically used for the preparation of the cathode however here it was used as an anodic binder in much lower concentration (5%) when compared to typically hydrophobic cathode material (20%). As the anode, carbon materials can significantly promote interfacial microbial colonisation and the formation of biofilm providing a conductive microenvironment for extracellular electron transfer an improving power density as a result [28]. A previous study reported adhesion of carbon black particles onto carbon veil matrix in a form of the microporous layer (MPL) with 2.2 times higher output against the control [33] and concluded that higher anodic surface area, through the microporous modification, had positive influence on bacterial growth on the anodes.

### 3.2 Electrochemical measurements

**Fig. 3:**
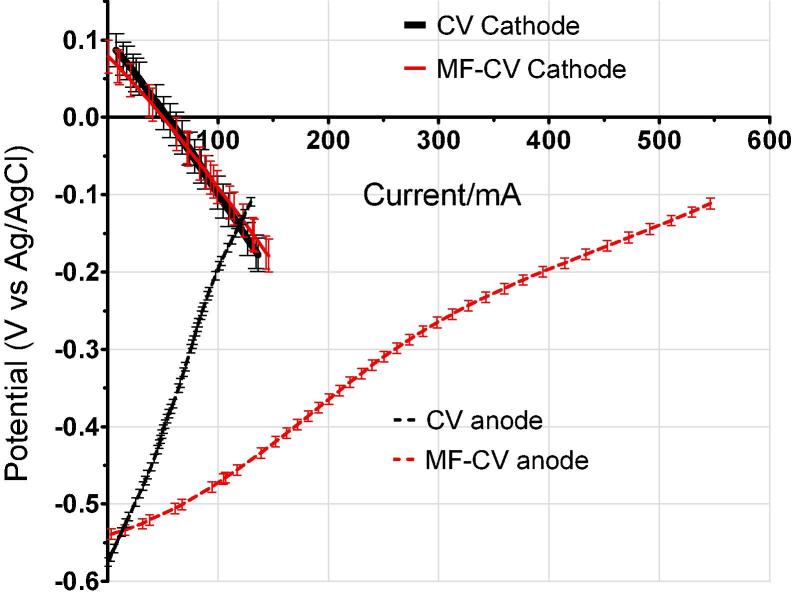
Linear sweep voltammetry of the cathodes and the anodes in tested CV and MF-CV modules. The data represents mean values based on two experimental runs (n = 2) with SD error bars.

Compared to the unmodified carbon veil anode, the MF-CV exhibited higher current output levels over an applied potential range during LSV. Fig. 3 shows that at the same potential of −0.15 V, the CV produced up to 117.0 mA SD ± 1.3 mA, whereas the MF-CV gave 479.3 mA ± 29.2 mA. Enhanced anodic activity of the MF-CV anode resulted in 4-fold increase in the anodic current. This higher current in MF-CV stack suggests that the modified anode facilitated higher specific surface area for the electroactive biofilm to adhere and provided increased reaction sites for substrate oxidation. This could promote direct electron transfer between the microorganisms as observed previously in granular activated carbon [37] and biochar electrodes [38]. The cathode LSV curves performed similarly in both stacks since they were identical in both set-ups. The mechanism of the enhancement of the anodic current can also be related to the micro structure of the activated carbon particles attached to the carbon veil scaffold visible in the SEM image that increases the specific surface area.

As can be seen in Fig. 3, with the improved anode performance, the cathode is limiting the system and it should be improved in the future.

**Fig. 4:**
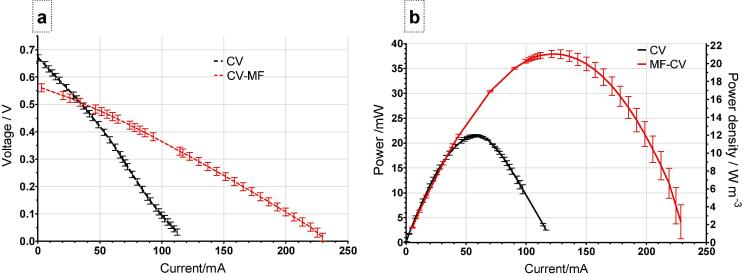
Polarisation experiment (a) and power curve (b). Data based on the mean data taken from two runs of polarisation sweeps.

Polarisation data showed that the open circuit value of the control stack was 671 mV and for the MF-CV was only 579 mV, however the current achieved at 20 mV was 117 mA for the control CV stack and 228 mA for the MF-CV (Fig. 4a). The maximum power point for MF-CV reached up to 37.9 mW and for the CV reached 21.4 mW (Fig. 4b). In terms of volumetric power density this is translating into 21.1 W m^−3^ and 11.9 W m^−3^ respectively and shows up to 77% improvement in power output, which is a similar increase in comparison to carbon black modification reported earlier in the individual set up [33]. The performance of the control is higher than the previously reported 6.8 W m^−3^ power density of a similar miniaturised stack and this was primarily due to differences in the type of ceramic material [15]. With the anodic modification reported in this work, the power density is improved further to 21.1 W m^−3^ which is 3 times higher than previously reported [15].

### 3.3 Long term operation

Since the purpose of the stack operation is power step-up to reach usable levels for practical implementation, this technology should sustain good outputs through prolonged periods. Currently, testing the technology in real-life scenarios and during prolonged periods is limited therefore it is mainly pursued in a lab-setting. Although this does not fully represent how the system would behave when implemented in real environments, it has the potential to provide important information about the set-up longevity in order to identify challenges associated with practical implementation. This particular work is concentrating on the modification of the anodic surface and its resilience to biofilm activity through extended periods of MFC stack operation.

**Fig. 5:**
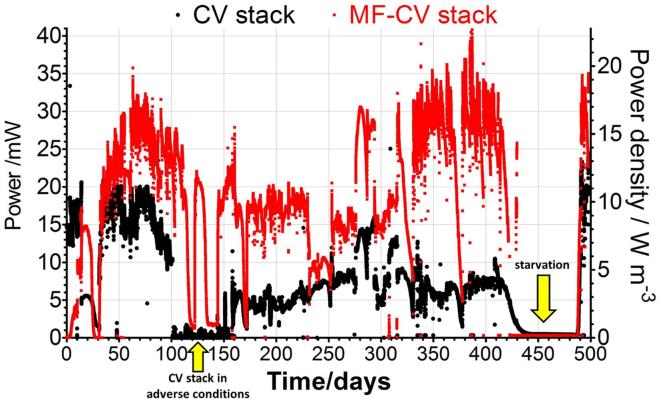
Power comparison between the stack equipped with the anode modification (MF-CV) and the control anode without the modification during 500 days (CV).

Fig. 5 shows the performance of both stacks during 500 days operation under batch-feeding regime, where the output of the CV stack stabilised at approximately 20 mW and at 33 mW for the MF-CV stack on day 70, which is consistent with the polarisation experiment. On day 100, the CV stack was removed from the logging device and connected to the larger stack (data not shown) running in adverse conditions, which may explain the underperformance continuing after day 150, when the CV stack was reconnected to the data logger (Fig. 5). The CV stack showed slow recovery, however did not reach the initial power level. The MF-CV stack on the other hand maintained good power output throughout, reaching up to 40.8 mW of power at day 386, whilst the control reached only 8.3 mW. Prolonged starvation period towards the end of the test lasted over two months and after that a fresh addition of urine restored the output of both stacks and the underperforming CV stack recovered back to the maximum performance levels. It demonstrates that starvation does not lead to deterioration of power as in previous stacked [39] and individual set-ups [40], but it actually can improve and restore the maximum power output of the stack that previously was underperforming. Throughout the long term study, it was observed that the cathodic wiring used for the parallel connections as well as stainless steel crocodile clips were corroded and needed to be replaced on multiple occasions. Corrosion and subsequent malfunction of the external wiring is a common issue in the long term prototype testing [41] and it remains a challenge.

### 3.4 Treatment

**Fig. 6:**
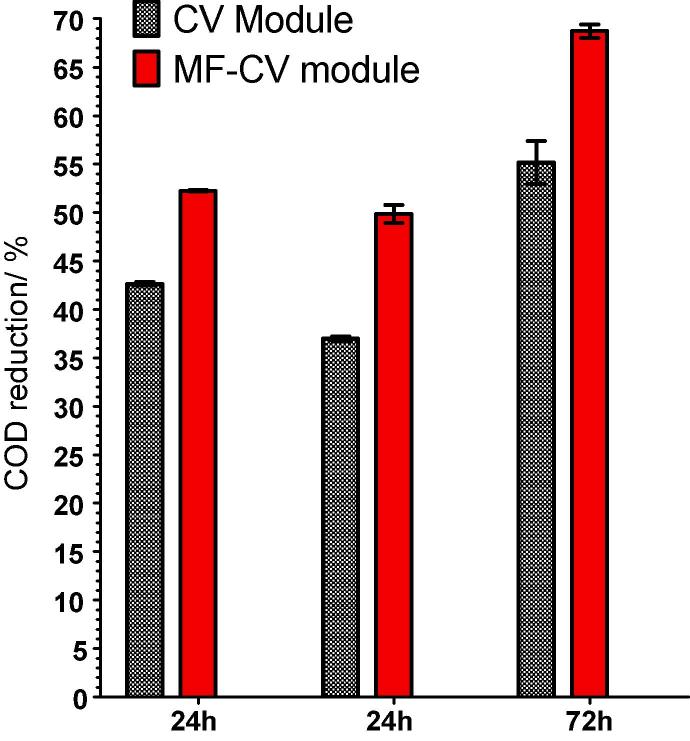
COD treatment performed three times with 24 h HRT during first and second test and 72 h during the third run.

COD reduction was of the order of 9.6%, 12.8% and 13.5% increase as a result of the anode modification, with the highest COD reduction being recorded during the longest HRT of 72 h where the CV stack showed 55.2% and the MF-CV 68.7% (Fig. 6). Treatment is directly related to the power output therefore the amount of biomass in the biofilm and the electrochemical activity of the electroactive biofilm in terms of produced current. In this case it might be due to both factors that play a role in the increase in treatment efficiency as the power output increase is evident in the electrochemical tests and the long term behaviour, which is the result of the surface modification that simply enhanced the specific surface area available for the biofilm growth onto additional –conductive layer on top of existing network of carbon fibres. The material characteristics clearly support the biofilm attachment, however the start-up time is in this case is slower due to the hydrophobicity of the added binder to the microporous ink. However, the power performance significantly increased. Activated carbon, as a versatile form of carbon with high surface area is currently used as support for the construction of efficient adsorbents for the removal of organic compounds [42] and dyes [43] and its properties can aid MFC power and treatment technology both in the anode and in the cathode half-cells.

### 3.5 Outlook

The cost of the carbon veil material used for this stack of 22 MFCs was approximately £3.17 whilst the activated carbon addition was £1.21, therefore the total cost of the modified anode used for a whole module was £4.38, which makes ∼£0.20 p per single MFC unit. This is a cost effective approach to the construction of MFC stacks in the future, which showed significant performance increase, applicability and robustness in long term operation. The challenges remain in finding optimum materials used for the peripheral electrical connections, which would be resistant to corrosion and fouling. Considering that approximately 2.5 billion people do not have access to proper sanitation, a significant potential for innovative sanitation technologies arises. More innovative ideas and development models are still required for resource recovery technologies from waste streams, such as urine, to be more widely adopted and accepted [4]. If MFC technology could be deployed for decentralised waste treatment or integrated into existing treatment plants, it has the potential to make the overall process more sustainable and energy-efficient [27]. Previously, a 20-module stack has been studied without the modified electrodes producing over 240 mW of power in total. Therefore, a 20-unit module of the modified electrode would be expected to give approximately 760 mW representing over a 3-fold increase in power production. This is an ongoing work pursued by our research centre and the modification will be integrated in all our future large-scale prototypes.

## 4 Conclusions

This work reports for the first time the implementation of simple and cost effective activated carbon modified anode electrodes employed in a stack of 22 small scale ceramic MFC units. Material characterisation revealed that activated carbon is forming a microporous structure available for the microbial attachment and the modification showed up to 37.9 mW of power which is 77% improvement in the stack power output and 13.5% increase in COD treatment. It exhibited a stable performance after 500 days and resilience to prolonged starvation periods. This novel anode modification is designed for low cost, large scale applications based on a modular approach, using miniaturised MFC units that efficiently treat urine and allow efficient energy recovery.

## CRediT authorship contribution statement

**Iwona Gajda:** Conceptualization, Data curation, Formal analysis, Investigation, Methodology, Visualization, Writing - original draft, Writing - review & editing. **John Greenman:** Conceptualization, Supervision, Writing - review & editing. **Ioannis Ieropoulos:** Conceptualization, Funding acquisition, Project administration, Resources, Supervision, Writing - review & editing.

## Declaration of Competing Interest

The authors declare that they have no known competing financial interests or personal relationships that could have appeared to influence the work reported in this paper.
